# Are multiple physical symptoms a poor prognostic factor or just a marker of depression severity? Secondary analysis of the GenPod trial

**DOI:** 10.1016/j.jad.2014.03.051

**Published:** 2014-07

**Authors:** Amy Green, Andrew Crawford, Katherine S. Button, Nicola Wiles, Tim J. Peters, David Nutt, Glyn Lewis

**Affiliations:** a Centre for Academic Mental Health, School of Social and Community Medicine, University of Bristol, United Kingdom; b School of Clinical Sciences, University of Bristol, United Kingdom; c Faculty of Medicine, Department of Medicine, Imperial College London, United Kingdom; d Division of Psychaitry, Faculty of Brain Sciences, University College, London, United Kingdom

**Keywords:** Depression, Antidepressants, Citalopram, Reboxetine, Physical symptoms

## Abstract

**Background:**

Using data from the GenPod trial this study investigates: (i) if depressed individuals with multiple physical symptoms have a poorer response to antidepressants before and after adjustment for baseline Beck Depression Inventory II (BDI-II); and (ii) if reboxetine is more effective than citalopram in depression with multiple physical symptoms.

**Methods:**

Linear regression models were used to estimate differences in mean BDI-II score at 6 and 12 weeks.

**Results:**

Before adjusting for baseline BDI-II, the difference in mean BDI-II score between no and multiple physical symptoms was 4.5 (95% CI 1.87, 7.14) at 6 weeks, 4.51 (95% CI 1.60, 7.42) at 12 weeks. After adjustment for baseline BDI-II, there was no evidence of a difference in outcome according to physical symptoms with a difference in mean BDI-II of 2.17 (95% CI −0.39, 4.73) at 6 weeks and 2.43 (95% CI −0.46, 5.32) at 12 weeks. There was no evidence that reboxetine was more effective than citalopram in those with multiple physical symptoms at 6 (*P=*0.18) or 12 weeks (*P=*0.24).

**Limitations:**

Differential non-adherence between treatment arms has the potential to bias estimates of treatment efficacy.

**Conclusion:**

Multiple physical symptoms predict response to antidepressants, but not after adjustment for baseline depression severity. Physical symptoms could be a marker of severe depression rather than an independent prognostic factor and depression should be considered in patients with multiple physical symptoms. Treatment with reboxetine conferred no advantage over citalopram in those with physical symptoms, and it is less well tolerated.

## 1 Introduction

Physical symptoms, which are distinct from those considered to be symptoms of depression (for example sleep and appetite disturbance) are common in depression (Haug et al., 2004; Bair et al., 2003). These types of symptoms (for example a change in bowel habit or pain) are often given as the presenting complaint as opposed to low mood (Bridges and Goldberg, 1985; Kirmayer et al., 1993; Keeley et al., 2004). Depressed patients are 3–7 times more likely to develop multiple physical symptoms than those who are not depressed (Hotopf et al., 1998). In many cases, no physical explanation for these symptoms is found and even when a disease state is present, the nature or degree of the symptoms may not correlate with the known pathology. A reduction in clinician׳s ability to detect depression has been shown with increasing levels of such physical symptoms (Bridges and Goldberg, 1985; Kirmayer et al., 1993; Bair et al., 2003). It has been estimated that 60% of previously undetected depression cases could have been identified if all primary care patients presenting with pain conditions were examined for possible depression (Katon, 1984). Patients in this group are therefore at risk of receiving an inaccurate diagnosis (Kirmayer et al., 1993; Bridges and Goldberg, 1985), are likely to use more healthcare resources (Bair, 2004; Fritzsche et al., 1999; Widmer and Cadoret, 1978) and are at risk of potential iatrogenic harm.

A ‘somatic depression’ has been proposed (Silverstein and Patel, 2011) that is more prevalent in women. The authors found that those who exhibited depression accompanied by multiple physical symptoms had a poorer response to antidepressants compared with the other depressed participants. Other studies have supported the theory that patients who have a depression with multiple physical symptoms have a poorer outcome in response to antidepressant treatment (Papakostas et al., 2003; Papakostas et al., 2008; Bair, 2004; Hoencamp et al., 1994) problem-solving therapy (Huijbregts et al., 2010) and collaborative care (Huijbregts et al., 2013). The Papakostas study of 2008 was a large (*n*=570), flexible dose, open-label trial of fluoxetine for major depressive disorder (MDD, as defined by DSM-IV). Using a self-report Symptom Questionnaire (Kellner, 1987) they found that the severity of somatic anxiety symptoms of MDD at baseline predicted a worse outcome with fluoxetine. The ARTIST study (Bair, 2004) also demonstrated that pain is a strong predictor of poor depression outcome.

The association between depression and pain becomes stronger as the severity of either condition increases (Bair et al., 2003). Therefore, an alternative theory is that patients with multiple physical symptoms have a more severe depression at baseline and therefore a poor prognosis. Denninger et al. (2006) reported that baseline somatic scores are related to baseline severity of depression. Severe depression at baseline predicts lower rates of remission with antidepressant treatment (Rush et al., 2008; Trivedi et al., 2006). This means that in order to understand the relationship between multiple physical symptoms in depression and prognosis with antidepressant treatment, the baseline severity of the depression must be taken in to account and adjusted for in the analysis. This has not been done in some of the studies that have predicted a poor outcome for depression with multiple physical symptoms (Silverstein and Patel, 2011; Papakostas et al., 2003).

Moderators are factors that predict differential treatment response. Identifying a moderator of antidepressant effect in patients with multiple physical symptoms has obvious clinical and economic value (Trusheim et al., 2007). Matching patients to treatments using particular patient characteristics (such as genetic polymorphisms or symptom profile) is called stratified or personalised medicine. Its benefits have been demonstrated with a number of anticancer medications (Trusheim et al., 2007). Psychiatrists commonly select antidepressants based on symptom profile (Zimmerman et al., 2004), but due to an absence of established clinical moderators (Simon and Perlis, 2010), there is limited evidence to inform this choice.

It is thought that brain mechanisms concerned with depression and pain both involve the monoamine projections from the midbrain (Bair et al., 2003). Noradrenaline is implicated in the aetiology of MDD and has been studied extensively (Ressler and Nemeroff, 1999). There is some evidence to suggest a relationship between noradrenaline and depression with physical symptoms (Fava, 2003). Dual acting agents (with an effect on serotonin and noradrenaline) have been reported as superior to selective serotonin re-uptake inhibitors (SSRIs) in treating the somatic symptoms associated with depression (Delgado, 2004; Fishbain, 2000). Kang et al. (2009) found that venlafaxine (a serotonin–noradrenaline re-uptake inhibitor) and mirtazipine (a noradrenergic and specific serotonergic receptor antagonist antidepressant) improved depressive symptoms in those with a diagnosis of MDD and somatic symptoms. Based on this evidence, the presence of physical symptoms could be a moderator of treatment in those with depression.

Stratified medicine aims to personalise treatment based on characteristics of an individual. One way of providing evidence to stratify antidepressant treatment is to compare two treatments in a group of patients and see if any particular characteristic moderates the relationship between treatment and outcome (18). The GenPoD trial looked at two potential moderators of response to antidepressants; (1) a polymorphism in the 5HTTLPR gene (the serotonin transporter gene) (Lewis et al., 2011) and (2) depression severity (Wiles et al., 2012). There was no evidence that either moderated treatment response. In this study, we used GenPod data to investigate the following hypotheses: (1) that those with multiple baseline physical symptoms do worse with antidepressant treatment at 6 and 12 weeks; and (2) that reboxetine is better than citalopram in treating patients with depression who have multiple baseline physical symptoms.

## 2 Methods

### 2.1 GenPoD trial

This is a secondary analysis of data from the GenPoD trial, whose trial protocol and main results are published elsewhere (Lewis et al., 2011; Thomas et al., 2008). In brief, GenPoD is a multi-centre Randomised Controlled Trial (RCT) conducted in Bristol, Birmingham and Newcastle, UK. Participants were patients aged 18–74 years referred to the trial by their General Practitioner (GP) following agreement to prescribe an antidepressant. Eligibility criteria included a diagnosis of ICD-10 depressive episode F32 from the Clinical Interview Schedule-Revised (CIS-R) (Lewis et al., 1992) and a Beck Depression Inventory II (BDI-II) (Beck At, 1961) score of ≥15. Exclusion criteria included having taken an antidepressant in the two weeks preceding baseline assessment and those who could not complete the self-administered scales. The GPs excluded those with medical contraindications to antidepressant treatment, those with psychosis, bipolar affective disorder or major substance or alcohol abuse. Chronic physical illness was not a contraindication to participation.

### 2.2 Randomisation

Participants were randomised to receive either reboxetine (4 mg twice daily) or citalopram (20 mg once daily) after giving informed consent. Randomisation was conducted using a computer-generated code, centrally administered and communicated by telephone. The randomisation was stratified by symptom severity (CIS-R <28 or≥28) and centre using variable block sizes. Neither participants nor researchers were blinded to allocation. The researcher gave the medication to the participant. Initially, those prescribed reboxetine were given a dose of 2 mg twice daily, which was increased to 4 mg twice daily after four days. Participants were advised to contact their GP if they wished to increase their dose.

### 2.3 Depression measures

The CIS-R was completed at baseline. This is a fully structured interview measuring common psychological symptoms present in the week prior to interview. It encompasses 14 symptom groups with the aim of identifying and characterising the symptoms of anxiety and depression (Lewis et al., 1992). The BDI-II measures the severity of depression and was completed at baseline, 6 and 12 weeks.

### 2.4 Exposure measures

The exposure variable of interest in this study was the presence of physical symptoms at baseline. This was measured using a modified form of the Toronto Side Effects Scale (Vanderkooy et al., 2002). For this purpose, the scale was used to measure physical symptoms at baseline before the participants had started the study medication. The modified scale used is included in the supplementary material (Supplementary Fig. 1). Symptoms that closely resembled symptoms of depression (agitation, daytime drowsiness and difficulty sleeping) were removed from the analysis since they are included in DSM-IV major depressive episode criteria (American Psychiatric Association, 2013). Gender-specific items (breast swelling, difficulty ejaculating and impotence) were also removed, leaving a total of 24 symptoms. Participants (*n*=601) were grouped into roughly equal quartiles by the number of physical symptoms they experienced; none (0 physical symptoms, *n*=161), few (1 physical symptom, *n*=126), several (2–3 physical symptoms, *n*=156) and multiple (4–16 physical symptoms, *n*=158) for 4–7 days of the week. Other measures used in the analysis included Social Support Score from the Psychiatric Morbidity among Adults Study (Sherbourne and Stewart, 1991), the AUDIT score for harmful alcohol consumption (Saunders et al., 1993) and the Short Form Health Survey- 12, a measure of physical and mental health status (Jenkinson et al., 1997).

### 2.5 Outcome measures

For the linear regression model, the outcome was BDI-II score at 6 and 12 weeks, a continuous variable. In order to make the results comparable to previous studies, a logistic regression model was also used, In this model, a binary variable of BDI-II of less than 10 was used at 6 weeks, to indicate recovery.

### 2.6 Statistical considerations

We used linear regression and logistic regression to examine the hypotheses of this study. Separate models were run at 6 and 12 weeks (linear regression model only). All models were adjusted for the trial design variables of baseline CIS-R score, medication allocation and recruitment centre. Further adjustment was made for the following potential confounders: baseline BDI-II score, adherence at 6 and 12 weeks, Social Support Score, life events score, gender, previous history of depression, age, centre, allocation, harmful alcohol use, baseline anxiety (using the Hospital Anxiety and Depression scale (Zigmond and Snaith, 1983) and chronic illness. The regression model included an interaction term between treatment (citalopram or reboxetine) and number of baseline physical symptoms to examine whether reboxetine is more effective than citalopram at treating depression with multiple baseline physical symptoms. As has been previously reported, age and life events were associated with missing data at 6 weeks (Wiles et al., 2012; Lewis et al., 2011). These factors were included in the regression model to investigate the impact of missing data on our findings. This method should address any bias under a missing at random assumption (Carpenter and Kenward, 2004). Analysis was performed on an intention-to-treat basis.

Details of the sample size calculations for the trial and the impact of the final recruitment figures on the power of the study are given elsewhere (Thomas et al., 2008). The GenPoD trial was adequately powered to address the original questions of the differential response to treatment and we used confidence intervals to guide our interpretation of the clinical importance of the results.

## 3 Results

### 3.1 Trial participation and follow-up

In the GenPoD trial, 601 participants were randomised to receive either citalopram (*n*=298) or reboxetine (*n*=303). At six weeks, 91% of participants (*n*=546) were included in the follow-up (citalopram: *n*=274; reboxetine: *n*=272). 81% completed the twelve week follow-up (*n*=486, citalopram: *n*=252; reboxetine: *n*=233). Some participants received an increase dosage of their antidepressant from their GP. For citalopram, 11 had their dosage increased to 30 mg/day, 33–40 mg/day and 11–60 mg (with one having an increase to an unknown amount). For reboxetine, 3 increased to 10 mg/day, 9–12 mg/day and one to 16 mg/day.

### 3.2 Distribution of physical symptoms in the trial population

**Fig. 1:**
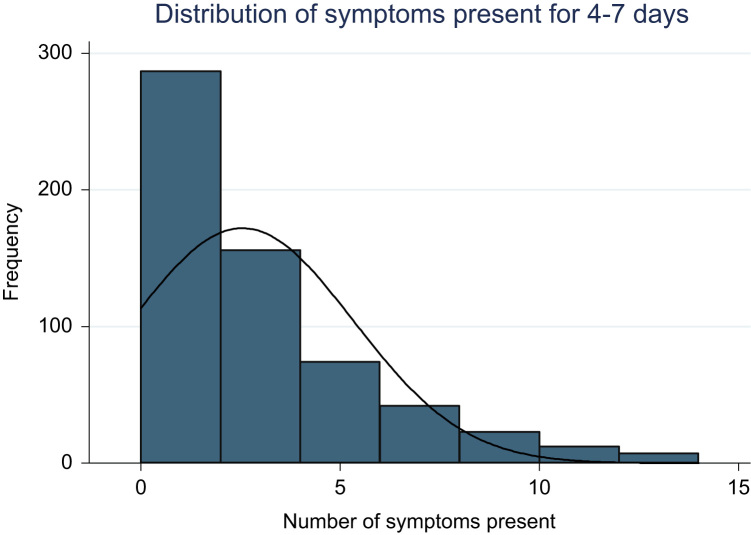
Distribution of physical symptoms present for 4–7 days/week.

Fig. 1 shows the distribution of physical symptoms present for 4–7 days in the previous week. The mean number of physical symptoms experienced for 4–7 days/week was 2.55 (standard deviation 2.77).

### 3.3 Baseline comparability of severity groups

Table 1 shows the baseline characteristics of the symptom quartiles. Individuals in the multiple physical symptoms group were more likely to be female (73% vs. 61%), report chronic illness (57% vs. 40%) and had a higher score on the BDI-II (37.94 vs. 29.67) than individuals with no physical symptoms at baseline.

### 3.4 Multiple physical symptoms and change in BDI-II score in response to antidepressants

**Fig. 2:**
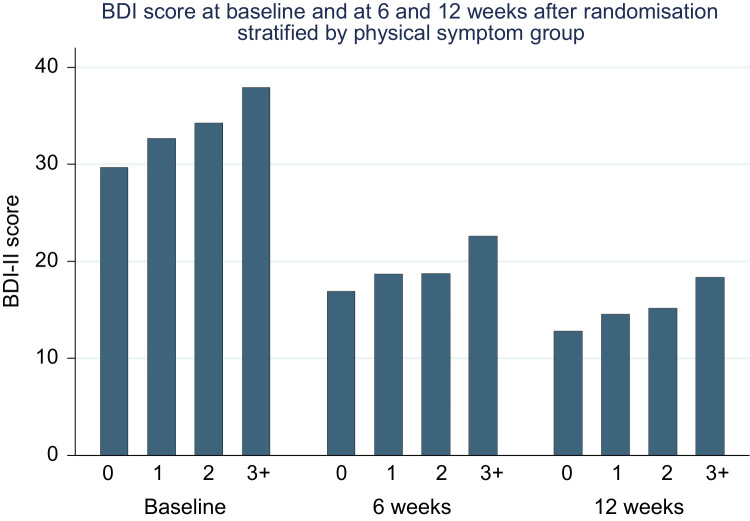
BDI-II scores at baseline, 6 and 12 weeks stratified by physical symptom group.

Table 2 shows adjusted differences in mean BDI-II score between no physical symptoms and few, several and multiple physical symptoms at 6 and 12 weeks for all treatments using the linear regression model. Before adjusting for baseline BDI-II, there was a large difference in mean BDI-II between no and multiple physical symptoms, at 6 (4.50 95% CI 1.87, 7.14) and 12 weeks (4.51 95% CI 1.60, 7.42). After adjusting for baseline BDI-II, the CIs included the null at 6 weeks (2.17 95% CI −0.39, 4.73) and 12 weeks (2.43 95% CI −0.46, 5.32). No other confounders had a substantial influence on the association. Fig. 2 shows BDI-II scores at baseline, 6 and 12 weeks stratified by physical symptom group.

The logistic regression model also produced no evidence to suggest that individuals reporting 3 or more physical symptoms at baseline were less likely to have a BDI score of less than 10 at 6 weeks than individuals with no physical symptoms at baseline (1.06, 95% CI 0.56, 2.00).

### 3.5 Citalopram vs. reboxetine in treating depression with multiple physical symptoms

We tested for an interaction between the physical symptom group and the randomised treatment, with BDI-II score (a continuous variable) at 6 and 12 weeks as the outcome. This provided no evidence that reboxetine was more effective than citalopram in those with multiple physical symptoms, at 6 (interaction *P=*0.18) and 12 weeks (interaction *P*=0.24). Table 3 shows mean BDI-II scores of the citalopram and reboxetine groups at 6 and 12 weeks by physical symptoms group, adjusted for trial design variables. When the analysis included factors associated with missing data (data not shown), the results were consistent with the main analysis.

## 4 Discussion

Multiple physical symptoms are associated with poorer response to antidepressants but we found no evidence for this relationship after adjusting for depression severity. This is supported by Denninger et al. (2006) who demonstrated that physical symptom scores at baseline did not predict reduction in HAM-D score with treatment, but opposes the findings of a number other studies (Silverstein and Patel, 2011, Papakostas et al., 2004; Bair, 2004; Keeley et al., 2004; Papakostas et al., 2003; Hoencamp et al., 1994).

There are some possible explanations for our findings. Multiple physical symptoms may represent severe depression and could be a sign of more severe illness. It could be that the severity of depression associated with multiple physical symptoms confers poor prognosis rather than the physical symptoms themselves. This is consistent with earlier theories on predictors of antidepressant response, whereby biological symptoms were used as a marker of depression severity (Delgado, 2004). It is possible multiple physical symptoms represent a misinterpretation of physical sensations, that is more likely to occur in depression and would usually be ignored by someone without depression. Therefore the more severe the depression, the more extreme the experience of physical symptoms.

An alternative theory is that increased physical symptoms are associated with an underlying physical condition that leads to poor health and consequently depression. Inflammatory processes have been implicated in this relationship (Raison et al., 2006). In our study, adjusting for chronic illness made little difference to the difference in mean BDI-II score between no and high scores at 6 weeks (4.16 95% CI 1.52, 6.81 vs. 4.50 95% CI 1.87, 7.14) and only a small difference at 12 weeks (3.74 95% CI 0.84, 6.65vs. 4.51 95% CI 1.60, 7.42).

Contrary to our findings, other large RCTs (Bair, 2004; Silverstein and Patel, 2011; Papakostas et al., 2003; Papakostas et al., 2008; Hoencamp et al., 1994; Keeley et al., 2004), have demonstrated that the presence of physical symptoms at baseline predicts an increased likelihood of non-response to an antidepressant. There are several possible explanations for this involving the method of data collection and/or data analysis. The most important difference is that some of these studies did not control for baseline severity of depression (Silverstein and Patel, 2011; Papakostas et al., 2003). Therefore, those individuals with a poor prognosis who had more somatic symptoms could have been more severely depressed in the first place.

We used a modified version of the Toronto Side Effects Scale (Vanderkooy et al., 2002) to measure physical symptoms. This is a broad screening tool encompassing different types of physical symptoms in multiple modalities. The Kellner symptom questionnaire (Kellner, 1987) used by Papakostas (Papakostas et al., 2008, 2003) and the SCL-90 (Derogatis et al., 1973) used byHoencamp et al. (1994) and Papakostas et al. (2008), include symptoms of depression such as poor appetite, and anxiety symptoms such as a choking feeling or hot spells. Keeley et al. (2004) have considered DSM-IV physical complaints associated with depression, generalised anxiety disorder and panic disorder as their measure of baseline physical symptoms. We excluded any symptoms that resembled well- established somatic symptoms of depression (for example, insomnia) and adjusted for anxiety in our analysis using a scale that avoided asking about the physical symptoms of anxiety (to prevent overcorrection) in case this was a confounder. Our data demonstrated that baseline anxiety was not a confounder but other studies have not done this (Silverstein and Patel, 2011; Papakostas et al., 2008). One possible explanation for these contradictory finding could be that the physical symptoms scale used in prior studies had an overlap with symptoms of depression.

Papakostas and colleagues (Papakostas et al., 2008) demonstrated an association between multiple physical symptoms and treatment response even after adjusting for baseline depression severity, and our study does not. It is difficult to fully explain this difference. As they have not presented their data before adjusting for baseline depression, we do not know what contribution this has made to the results. This makes their results hard to interpret. The association they found between baseline somatic scores and treatment response (OR 0.96 95% CI 0.94–0.98, *P=*0.002) was statistically significant. Since a 3-point difference in the BDI-II score corresponds to a minimally important clinical difference (NICE guidelines, 2004), this difference may not have clinical relevance. Similarly, our 95% CIs indicate that individuals reporting multiple physical symptoms at baseline may score up to 4 points higher on the BDI at 6 (2.17, 95% CI −0.39, 4.75 and 12 weeks (2.43, 95% CI −0.46, 5.32), which may also not be of clinical relevance.

There was no evidence of a difference between citalopram and reboxetine in treating depression with multiple baseline physical symptoms and no evidence that physical symptoms are a moderator of the antidepressant effect of SSRIs and NARIs. Using data from the GenPod trial, Wiles et al. (2012) also found that treatment with NARIs did not confer any advantage over SSRI treatment for outcome in those with more severe depression. Therefore, noradrenergic antidepressants do not appear to be superior to serotonergic antidepressants in treating depression in those with multiple physical symptoms or those with more severe depression in this primary care population. Since reboxetine was associated with more adverse effects and more drop-outs (Cipriani et al., 2009; Crawford et al., 2014), this suggests that other agents should take preference over a NARI.

The GenPoD trial is a suitable data set to address both our hypotheses. Data were collected from primary care patient with a diagnosis of severe depression (mean baseline BDI was 33.7). It compared two different classes of antidepressants to see whether a specific patient characteristic moderates the relationship between treatment type and outcome. This is an appropriate way of testing for a moderator effect (Simon and Perlis, 2010). Secondary analysis testing for moderation can suffer from low power (Brookes et al., 2004; Brown, 1988), but since the GenPod study was originally designed to test for an interaction effect, this was not a major issue in this case, and indeed in the event the confidence intervals were relatively narrow.

## 5 Limitations

There are a few limitations to this study. Follow-up data were available for 81% of trial participants at 12 weeks. Even a small amount of missing data has the potential to introduce bias; however, adjustment for factors associated with missing data produced results consistent with our main analysis.

More participants allocated to reboxetine discontinued antidepressant treatment than those allocated to citalopram (36% vs. 17% at 6 weeks) (Crawford et al., 2014). This is consistent with the meta-analysis findings of Cipriani et al. (2009). Non-adherence to treatment, particularly differential non-adherence between treatment arms, has the potential to bias estimates of treatment efficacy. The reboxetine group suffered more adverse effects, but there was not strong evidence of an association between adverse effects and drop-out. 42% of participants had discontinued their medications at 12 weeks. A lack of blinding may have introduced a source of bias as the participants knew which drug they were receiving. However, the participants would have required strong preconceptions on the effectiveness of each drug and so this is unlikely to have influenced the results. Despite the relatively large sample size it is possible that we did not have sufficient power to detect interactions, which may have resulted in type II errors; however, we have used the confidence intervals obtained to guide interpretation in respect of potential clinical importance.

Finally, trial participants themselves are often a highly selected group, which can lead to issues of generalisability. On the other hand, participants were recruited from primary care, and the study included those with chronic physical illness, which is likely to increase the generalisability of the findings. Moreover, the analysis adjusted for chronic illness, baseline depression and anxiety as well as other potential confounders.

## 6 Conclusions

Multiple baseline physical symptoms appear to predict poor response to antidepressants, but not after adjustment for depression severity. Multiple physical symptoms may represent severe depression rather than a poor prognostic factor. Clinicians should ask about mood symptoms and explore psychosocial issues in patients with multiple physical symptoms. If considering a diagnosis of depression in someone with multiple physical symptoms, clinicians should treat it as a moderate to severe depression. This is essential in order to avoid inappropriate investigation and treatment of physical symptoms, which could lead to iatrogenic harm. Reboxetine does not confer any advantage over citalopram in this group, and it is less well tolerated. Since there is no evidence that physical symptoms are a moderator of treatment in patients with multiple physical symptoms when a clinician chooses an antidepressant they should take account of the side effect profile, rather than depend on physical symptoms.

## Role of funding source

This work was supported by the Medical Research Council United Kingdom of Great Britain and Northern Ireland (Grant ref: G0200243).

## Conflict of interest

No conflict declared.

## Figures and Tables

**Table 1:** Characteristics of participants, according to baseline physical symptoms.

	**Overall (*****N*****=601)**	**None (*****N*****=161)**	**Few (***N*=**126)**	**Several (*****N*****=156)**	**Multiple (*****N*****=158)**
**Mean age (SD)**	38.82 (12.35)	38.16 (11.05)	39.88 (12.65)	38.21 (12.31)	39.27 (13.40)
**N (%) female**	408 (67.87)	98 (60.87)	77 (61.11)	119 (76.28)	114 (72.15)
**N (%) CIS-R >=28**	394 (65.56)	73 (45.34)	75 (59.52)	105 (67.31)	141 (89.24)
**Mean BDI tot (SD)**	33.67 (9.70)	29.67 (8.37)	32.68 (8.70)	34.27 (9.33)	37.94 (10.17)
**Mean life events score (SD)**	1.67 (1.30)	1.44 (1.27)	1.42 (1.16)	1.81 (1.42)	2.00 (1.48)
**Mean social support score (SD)**	12.02 (3.80)	12.51 (3.50)	11.80 (3.99)	11.74 (3.77)	11.96 (3.94)
**N (%) taking citalopram**	298 (49.60)	81 (50.31)	58 (46.03)	84 (53.85)	75 (47.47)
**N (%) in full time work**	243 (40.43)	70 (43.48)	55 (43.65)	56 (35.90)	62 (39.24)
**N (%) with no previous depression**	167 (27.80)	49 (30.63)	33 (26.19)	42 (26.93)	41 (25.95)
**Mean anxiety symptoms on CIS-R (SD)**	2.49 (1.47)	2.11 (1.50)	2.24 (1.32)	2.37 (1.51)	2.94 (1.41)
**Mean AUDIT score (alcohol use) (SD)**	4.01 (3.70)	4.09 (3.04)	4.00 (3.89)	4.19 (3.88)	3.76 (3.85)
**N (%) chronic illness**	288 (47.92)	64 (39.75)	53 (42.06)	81 (51.92)	90 (56.96)
**Mean HAD anxiety score (SD)**	13.22 (3.57)	12.06 (3.42)	12.59 (3.37)	13.19 (3.65)	14.95 (3.12)
**Mean HAD depression score (SD)**	12.6 (4.00)	11.55 (3.76)	12.12 (3.71)	12.49 (4.02)	14.16 (4.00)
**Number of Caucasians (%)**	575 (95.67)	158 (98.14)	115 (91.27)	152 (97.44)	150 (94.94)

SD (standard deviation), BDI (Beck Depression Inventory), CIS-R (Clinical Interview Schedule-Revised), HAD (Hospital Anxiety and Depression scale).

**Table 2:** Differences in BDI-II score between no physical symptoms and few, several and multiple physical symptoms at 6 and 12 weeks for all treatments.

	**None (*****N*****=161)**	**Few (*****N***=**126)**	**Several (*****N*****=156)**	**Multiple (*****N*****=158)**
**Data at 6 weeks**				
Unadjusted difference in BDI score at 6 weeks^a^	reference	1.35 (−1.28, 3.98)	1.31 (−1.23, 3.84)	4.50 (1.87, 7.14)
Adjusted difference in BDI score at 6 weeks^b^		0.53 (−1.97, 3.03)	−0.19 (−2.61, 2.24)	2.17 (−0.39, 4.73)
Adjusted for history of depression		1.32 (−1.32, 3.95)	1.25 (−1.29, 3.79)	4.46 (1.82, 7.10)
Adjusted for gender		1.28 (−1.34, 3.90)	0.96 (−1.59, 3.52)	4.16 (1.50, 6.82)
Adjust for life events		1.36 (−1.28, 3.99)	1.24 (−1.31, 3.78)	4.40 (1.74, 7.06)
Adjust for chronic illness		1.36 (−1.26, 3.97)	1.23 (−1.30, 3.97)	4.16 (1.52, 6.81)
Adjusted for social support		1.10 (−1.49, 3.68)	0.94 (−1.55, 3.44)	4.33 (1.74, 6.92)
Adjusted for HAD- anxiety		1.36 (−1.24, 3.96)	1.13 (−1.38, 3.63)	3.64 (1.00, 6.28)
Adjusted for adherence at 6 weeks		1.24 (−1.37, 3.71)	1.19 (−1.34, 3.71)	4.47 (1.85, 7.10)
Adjusted for CISR anxiety		0.57 (−2.17, 3.33)	1.21 (−1.49, 3.92)	3.97 (1.32, 6.61)

**Data at 12 weeks**				
Unadjusted difference in BDI score at 12 weeks^a^		1.51 (−1.42, 4.44)	1.86 (−0.93, 4.66)	4.51 (1.60, 7.42)
Adjusted difference in BDI score at 12 weeks^b^		0.78 (−2.05, 3.61)	0.67 (−2.05, 3.40)	2.43 (−0.46, 5.32)
Adjusted for history of depression		1.32 (−1.59, 4.24)	1.63 (−1.16, 4.41)	4.25 (1.35, 7.15)
Adjusted for gender		1.38 (−1.55, 4.30)	1.46 (−1.36, 4.28)	4.13 (1.19, 7.06)
Adjust for life events		1.45 (−1.46, 4.37)	1.71 (−1.07, 4.50)	3.99 (1.06, 6.93)
Adjust for chronic illness		1.56 (−1.33, 4.45)	1.61 (−1.16, 4.37)	3.74 (0.84, 6.65)
Adjusted for social support		1.30 (−1.58, 4.18)	1.35 (−1.41, 4.11)	4.16 (1.29, 7.04)
Adjusted for HAD- anxiety		1.56 (−1.34, 4.46)	1.65 (−1.12, 4.42)	3.70 (0.77, 6.63)
Adjusted for adherence at 12 weeks		1.56 (−1.37, 4.50)	1.88 (−0.92, 4.68)	4.58 (1.66, 7.51)
Adjusted for CISR anxiety 12 weeks		0.62 (−2.44, 3.69)	2.23 (−0.77, 5.23)	3.55 (0.61, 6.48)

BDI (Beck Depression Inventory), CIS-R (Clinical Interview Schedule-Revised), HAD (Hospital Anxiety and Depression scale).

a Adjusted for trial variables.

b Adjusted for baseline BDI-II.

**Table 3:** Mean BDI-II scores of the citalopram and reboxetine groups as randomised, at 6 and 12 weeks by physical symptoms group, adjusted for trial variables.

	**Overall (*****N*****=601)**	**None (*****N*****=126)**	**Few (*N*=131)**	**Several (*****N*****=148)**	**Multiple (*****N*****=196)**
BDI at 6 weeks for citalopram (SD)	18.87(10.82)	16.88 (9.37)	18.13 (9.88)	17.64 (11.00)	22.89 (11.92)
BDI at 6 weeks for reboxetine (SD)	19.58 (11.47)	16.95 (10.05)	19.13 (10.96)	20.00 (11.11)	22.26 (11.94)
Adjusted difference between citalopram and reboxetine at 6 weeks (SE)	NA	1.01 (1.42)	0.75 (1.88)	3.14 (1.91)	1.11 (1.83)
BDI at 12 weeks for citalopram (SD)	15.38 (11.47)	13.62 (9.43)	14.58 (9.88)	13.24 (10.62)	20.29 (14.17)
BDI at 12 weeks for reboxetine (SD)	15.09 (11.29)	11.87 (9.12)	14.51 (9.50)	17.41(12.65)	16.50 (12.50)
Adjusted difference between citalopram and reboxetine at 12 weeks (SE)	NA	0.66 (1.54)	0.07 (1.88)	4.9 (2.10)	3.77 (2.23)

BDI (Beck Depression Inventory), SD (standard deviation), SE (standard error).
