# Supplementary material for: Are multiple physical symptoms a poor prognostic factor or just a marker of depression severity? Secondary analysis of the GenPod trial
Source: J Affect Disord. 2014 Jul;163:40–6. doi: 10.1016/j.jad.2014.03.051 (PMC4315809; doi:10.1016/j.jad.2014.03.051)
Supplement: Supplementary file 1 — Supplementary Data [file mmc1.pdf]

Supplementary material

Figure 1. Modified version of the Toronto Side Effects Scale (symptoms experienced for 4-7 days).

1. Back pain
2. Chest pain
3. Stiffness in your arms or legs
4. Headaches
5. Sore throat
6. Tenderness of the glands in your neck
7. A rapid heart beat
8. Tremor
9. Dry mouth
10. Excessive sweating
11. Tingling in your limbs, fingers or toes
12. Stomach pains
13. Constipation
14. Diarrhoea
15. Felt sick or nauseous
16. Noticed changes in the way your food tastes
17. Light headedness or dizziness
18. Shortness of breath at rest
19. Ringing in the ears
20. Increased sensitivity to light or noise
21. Difficulty or pain passing urine

22. Passed urine more often
23. Skin rash or irritation
24. Hot flushes
